# Trends and Outcomes of Lower Limb Amputation in Patients With Coronary Artery Disease

**DOI:** 10.7759/cureus.79054

**Published:** 2025-02-15

**Authors:** Abdul Rasheed Bahar, Yousef Alsmairat, Yasemin Bahar, Mohamed S Alrayyashi, Mobeen Z Haider, Prakash Upreti, Ali Al-Ramadan, Olayiwola Bolaji, Mohammad Hazique, M. Chadi Alraies

**Affiliations:** 1 Internal Medicine, Wayne State University, Detroit Medical Center, Detroit, USA; 2 Cardiology, West Virginia University, Morgantown, USA; 3 Internal Medicine, BronxCare Health System, Bronx, USA; 4 Internal Medicine, University of Maryland Capital Regional Medical Center, Largo, USA; 5 Internal Medicine, Nuvance Health, Vassar Brothers Medical Center, Poughkeepsie, USA; 6 Cardiology, Wayne State University, Detroit Medical Center, Detroit, USA

**Keywords:** coronary artery disease, cost efficiency, healthcare outcomes, lower limb amputation, national inpatient sample database

## Abstract

Background and aim

Coronary artery disease (CAD) is a leading cause of morbidity and mortality in the United States, with peripheral arterial disease (PAD) and lower limb (LL) amputation contributing to poor cardiovascular outcomes. While previous studies have identified the link between CAD and PAD-related amputations, data on short-term in-hospital outcomes remain limited. This study aimed to compare in-hospital mortality and complications between CAD patients undergoing LL amputation and those without it.

Methods

We conducted a retrospective cohort study using the Nationwide Inpatient Sample (NIS) from 2016 to 2021, identifying CAD patients with and without LL amputation via International Classification of Diseases, 10th Revision (ICD-10) codes. Propensity score matching (PSM) was performed using a 1:1 nearest-neighbor algorithm to minimize selection bias, adjusting for demographics, comorbidities, and hospital characteristics. The primary outcome was in-hospital all-cause mortality, while secondary outcomes included acute heart failure, cardiogenic shock, acute kidney injury (AKI), major adverse cardiac and cerebrovascular events (MACCE), and healthcare resource utilization.

Results

A total of 31,379,939 CAD patients were identified, with 119,320 (0.4%) undergoing LL amputation. After propensity score matching (PSM), 23,261 patients were included in each group. The LL amputation cohort exhibited significantly higher in-hospital mortality (5.5% vs. 3.3%, p<0.001), cardiac arrest (2.3% vs. 1.4%, p<0.001), acute kidney injury (AKI) (29.3% vs. 26.8%, p<0.001), and acute limb ischemia (5.2% vs. 0.4%, p<0.001). Conversely, CAD patients without amputation had higher rates of acute heart failure (18.3% vs. 10.7%, p<0.001), major adverse cardiac and cerebrovascular events (MACCE) (22.5% vs. 12.2%, p<0.001), and percutaneous coronary intervention (6.7% vs. 0.9%, p<0.001). The length of stay and total hospital charges were significantly higher in the amputation group (10 days vs. four days; $26,590 vs. $11,686, p<0.001).

Conclusion

Lower limb amputation in CAD patients is associated with increased in-hospital mortality, cardiac complications, and healthcare resource utilization. These findings underscore the need for early intervention strategies targeting PAD progression and comprehensive perioperative cardiovascular risk management in amputees. Future research should focus on optimizing revascularization approaches, rehabilitation programs, and tailored preventive measures to improve outcomes in this high-risk population.

## Introduction

Coronary artery disease (CAD), which is mainly due to atherosclerosis in the coronary arteries, is considered the leading cause of death among both men and women in the United States [1,2]. Atherosclerotic plaque development was once thought to be solely related to dyslipidemia. However, it is now understood to involve a complex interplay of factors, including endothelial dysfunction, oxidative stress, inflammation, vascular smooth muscle activation, lipid disturbances, platelet aggregation, and thrombosis [3].

Multiple risk factors, such as hypertension, dyslipidemia, diabetes mellitus, chronic kidney disease, age, sex, lifestyle, smoking, diet, obesity, and family history influence the pathophysiology of CAD [4]. Peripheral arterial disease (PAD) shares a similar pathophysiology with CAD and is associated with similar risk factors. Patients with PAD are classified as being at high cardiovascular risk [4]. CAD and PAD are both manifestations of systemic atherosclerosis, leading to similar risk factors and adverse cardiovascular outcomes. PAD is a known predictor of poor cardiovascular prognosis, and severe cases may necessitate amputation due to critical limb ischemia [5].

Studies have shown that PAD is linked to a high mortality rate. A meta-analysis by Agnelli et al. 2020, which included 124 studies, reported an all-cause and cardiovascular mortality rate of 70 per 1,000 person-years. The pooled event rate for significant amputations was 70 per 1,000 person-years [6]. Studies show that up to 50% of major amputations occur in individuals with documented CAD or PAD. Moreover, post-amputation mortality rates remain alarmingly high, with a one-year mortality rate reaching 37.7% and five-year mortality exceeding 70%. This is often due to cardiovascular complications such as myocardial infarction, heart failure, and arrhythmias [7,8].

Lower extremity amputation has a significant impact on the quality of life of affected patients and is associated with a high mortality rate. A meta-analysis of 61 studies involving 36,037 patients found a pooled one-year mortality rate of 37.7% following non-traumatic lower extremity amputation [8]. Other studies have reported a 30-day mortality rate ranging from 22% to 30% after non-traumatic lower extremity amputation [9,10] The disproportionate burden of CAD among amputees and limited data on short-term in-hospital outcomes necessitate further investigation. Identifying differences in mortality, complications, and healthcare utilization will help optimize perioperative management and develop targeted interventions to improve outcomes. Given the close relationship between CAD and PAD, this study aimed to compare the mortality rate and other outcomes in CAD patients who undergo lower extremity amputation versus those who do not.

## Materials and methods

Data source

This study is a retrospective cohort analysis of patients admitted to hospitals across the United States with coronary artery disease (CAD). The data were drawn from the Nationwide Inpatient Sample (NIS) database from 2016 to 2021, part of the Healthcare Cost and Utilization Project (HCUP) sponsored by the Agency for Healthcare Research and Quality (AHRQ) [11]. The NIS is the largest publicly available all-payer inpatient healthcare database in the United States, providing nationally representative estimates of inpatient utilization, access, outcomes, cost, and quality. A 20% random sample of patients within each stratum is selected, and their demographic information, diagnoses, and resource utilization are entered into the database. Each discharge is then weighted (weight = total number of discharges from all U.S. acute care hospitals/number of discharges in the 20% sample) to ensure the NIS is nationally representative. It contains a primary discharge diagnosis and can have up to 39 secondary diagnoses including comorbid conditions and complications during hospitalization. The database also provides information regarding patient demographic information, resource utilization such as length of stay (LOS) and total hospitalization charges, hospital characteristics, insurance status, in-hospital outcomes, and primary and secondary procedures, all identified using codes from the International Classification of Diseases, 10th Revision, Clinical Modification (ICD-10-CM) [11,12]. The present study follows the methodological design checklist proposed by Khera et al. to address common study design errors in NIS-based research [13]. Since the NIS is a publicly available, anonymized national database with prior ethical approval from the AHRQ, this study was exempt from institutional review board (IRB) approval.

Study population

We included patients with a principal diagnosis of CAD utilizing the NIS database from January 1, 2016, to December 31, 2021. Patients were excluded if they were less than 18 years old. Patients with overlapping diagnoses (e.g., trauma-related amputations) were excluded to maintain the specificity of our CAD-related amputation cohort. Lower limb (LL) amputation was defined as right or left-sided above-knee amputation (AKA) or below-knee amputation (BKA). Patients were then divided into the following two cohorts: those with and without LL amputation. The ICD-10-CM codes were validated through an independent review by two authors with minimal interobserver variability (<5%), and discrepancies were resolved through consultation with a third author and further code iteration as needed. The ICD-10 codes used in this study were compared to those used in previously published research papers [14]. The list of ICD-10-CM diagnoses and procedural codes used in this study are presented in the tables in the appendix.

Definitions and outcomes

**Figure 1:**
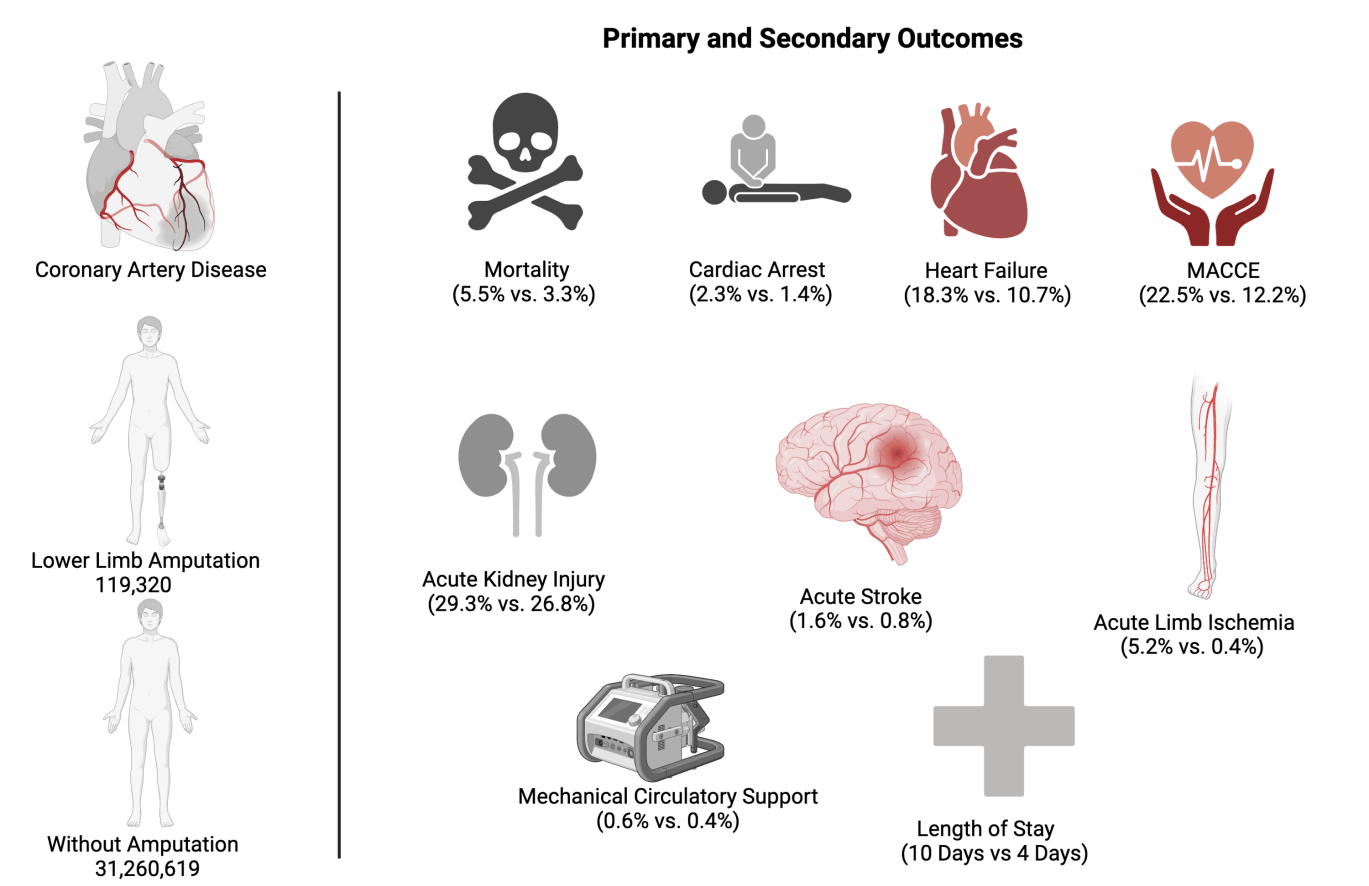
Trends and outcomes of lower limb amputation in patients with coronary artery disease. All figures are original to this study and created by the authors. MACCE: major adverse cardiac and cerebrovascular events

The primary outcome was in-hospital all-cause mortality. Secondary outcomes were as follows: (i) lower limb specific outcomes such as acute limb ischemia (ALI), intravascular ultrasound (IVUS), percutaneous revascularization, thrombectomy, and fasciotomy, (ii) morbidity as measured by the development of acute heart failure, cardiogenic shock, sudden cardiac arrest (SCA), acute stroke, major adverse cardiac and cerebrovascular events (MACCE), and acute kidney injury (AKI), (iii) resource utilization as measured by LOS and total hospitalization charges and costs (Figure 1). MACCE was defined as a composite of acute myocardial infarction, cardiogenic shock, cardiac arrest, and in-hospital mortality, in accordance with prior research [15,16]. Various potential confounding factors were identified and accounted for during the analysis, including the patient’s age, sex, race, income based on ZIP code, Charlson Comorbidity Index, insurance status, bed size, hospital teaching status, hospital urban location, and geographic region. All the abovementioned outcomes were identified using ICD-10-CM diagnoses and procedure codes (tables in the appendix). LOS, total hospitalization charges, and patient demographics were directly obtained from the NIS database. Total hospital charges reflect the amount a patient was billed for the entire hospital stay, but they do not reflect the actual cost of care. The HCUP provides data with hospital-specific cost-to-charge ratios based on inpatient costs across all payers [17]. Using this information, total hospital costs were calculated by multiplying the hospital charges by the corresponding cost-to-charge ratio. All hospitalization costs and charges were adjusted for inflation over time using the hospital services part of the consumer price index and are represented in 2021 US dollars [18]. Patient demographics included age (in years), sex, race (categorized as Caucasian, Black, Hispanic, Asian or Pacific Islander, Native American, and other), primary expected payer (Medicare, Medicaid, private insurance, and uninsured), hospital bedside (small, medium, and large), teaching status, hospital region (Northeast, Midwest, South, and West), and urban location [19]. The comorbidity burden was assessed using claims-based disease-specific refinements matching translation to the ICD-10 and flexibility (Claims-based, Disease-Specific Refinements, Matching Translation to ICD-10, Flexibility-Charlson Comorbidity Index {CDMF-CCI}) model [20].

Statistical analysis

All statistical analyses were performed using Stata statistical software version 18 (College Station, TX: Stata Corp. LLC), with a two-sided p<0.05 considered statistically significant. The NIS is built on a complex sampling design incorporating stratification, clustering, and weighting. Stata software enables analysis to generate nationally representative, unbiased results, variance estimates, and p-values. All analyses utilized weighted NIS data in compliance with the HCUP guidelines [21]. Each hospital admission was assigned a discharge weight to estimate national in-hospital outcomes to account for clustering, weighting, and stratification to represent the broader United States population accurately. The following variables had missing data: day and month of admission, age, race, sex, insurance status, and elective versus non-elective admission. These missing data were addressed and treated using multiple imputations by predictive mean matching, preserving the distribution and variability of the observed data while incorporating the relationships among variables [22,23].

Descriptive statistics were used to summarize the demographic characteristics of the study population. Continuous variables were tested for normality of distribution using the Shapiro-Wilk test and reported as means with standard deviations (SD) or medians with interquartile ranges (IQR), depending on their distribution. Categorical variables were expressed as absolute counts with corresponding percentages. Group comparisons were performed using the Wilcoxon signed-rank test for non-normally distributed continuous variables and Pearson’s chi-square test for categorical variables.

Trends in CAD hospitalizations, LL amputation, demographic characteristics, and study outcomes over time were analyzed using the Jonckheere-Terpstra and Cuzick’s trend tests. A two-stage multivariable logistic regression model was used to identify independent predictors of study outcomes. In the first stage, univariate logistic regression was performed for all potential predictors, and variables with p<0.05 were selected for inclusion in the final model. In the second stage, these significant variables were incorporated into a multivariable model to evaluate their independent associations while adjusting for confounders. The analysis accounted for patient-level factors, medical comorbidities, socioeconomic status, and hospital-level characteristics. To address within-hospital clustering, a mixed-effects model was applied, treating hospital-level clustering as a random effect. Propensity score matching (PSM) was performed using 1:1 nearest-neighbor matching with a caliper of 0.2 standard deviations to minimize selection bias. Propensity scores were derived from a logistic regression model including demographics, hospital characteristics, and comorbidities. Balance was assessed using standardized mean differences (SMD<0.1 considered adequate), and post-matching outcomes were compared using Pearson’s chi-square and Wilcoxon rank-sum tests.

## Results

**Figure 2:**
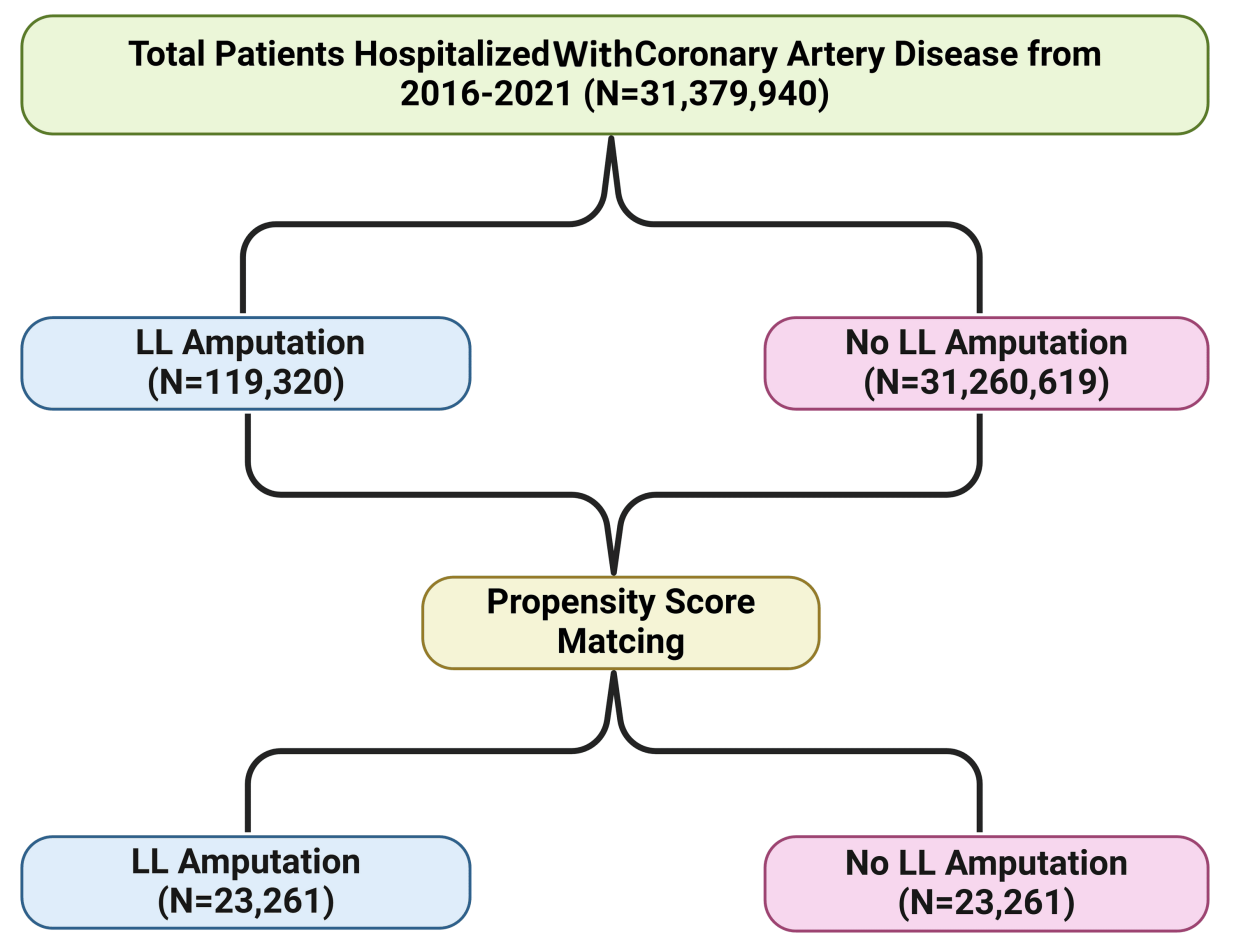
Flow diagram of included study population. All figures are original to this study and created by the authors. LL: lower limb

We performed a retrospective analysis of the national inpatient sample database from 2016 to 2021 to identify patients with coronary artery disease and classified the patients into those with and without amputation. A multivariate analysis using logistic regression was done. We identified a total of 31,379,939 patients with CAD that were classified into 119,320 (0.4%) patients with coronary artery disease and amputation and 31,260,619 (99.6%) patients with coronary artery disease without amputation (Figure 2).

Patient and hospital characteristics

As tabulated in Table 1, 68.2% of the patients with coronary artery disease with amputation arm were males whereas 55.4% were in the CAD arm.

**Table 1:** Baseline characteristics of CAD patients with and without LL amputation. MI: myocardial infarction; PCI: percutaneous coronary intervention; CAD: coronary artery disease; LL: lower limb Descriptive statistics were used for comparing baseline characteristics and comorbidities. Continuous variables were compared using the Student's t-test in the unadjusted analyses and are presented as mean±SD. P<0.05 was considered statistically significant.

Variables		CAD+LL amputation	CAD only	p-Value
N		119,320 (0.4%)	31,260,619 (99.6%)	
Demographics				
Sex	Male	81,350 (68.2%)	17,314,864 (55.4%)	<0.001
	Female	37,970 (31.8%)	13,945,755 (44.6%)	
Age (year), mean (SD)		66.75 (11.48)	70.78 (13.03)	<0.001
Race	White	71,470 (61.5%)	22,248,082 (73.0%)	<0.001
	Black	26,700 (23.0%)	4,267,764 (14.0%)	
	Hispanic	13,010 (11.2%)	2,373,974 (7.8%)	
	Asian or Pacific Islander	1,605 (1.4%)	666,360 (2.2%)	
	Native American	1,110 (1.0%)	165,225 (0.5%)	
	Other	2,410 (2.1%)	739,084 (2.4%)	
Hospital metrics	Length of stay, median (IQR)	10 (7-16)	4 (2-6)	<0.001
Region of hospital	Northeast	19,100 (16.0%)	6,039,622 (19.3%)	<0.001
	Midwest	27,175 (22.8%)	7,438,182 (23.8%)	
	South	54,670 (45.8%)	12,608,544 (40.3%)	
	West	18,375 (15.4%)	5,174,272 (16.6%)	
Location/teaching status of hospital	Rural	8,045 (6.7%)	2,888,533 (9.2%)	<0.001
	Urban non-teaching	19,580 (16.4%)	6,621,898 (21.2%)	
	Urban teaching	91,695 (76.8%)	21,750,188 (69.6%)	
Bed size of hospital	Small	19,080 (16.0%)	6,541,717 (20.9%)	<0.001
	Medium	34,030 (28.5%)	9,187,006 (29.4%)	
	Large	66,210 (55.5%)	15,531,896 (49.7%)	
Primary insurance	Medicare	85,895 (72.1%)	22,420,752 (71.8%)	<0.001
	Medicaid	13,975 (11.7%)	2,761,079 (8.8%)	
	Private insurance	14,430 (12.1%)	4,551,859 (14.6%)	
	Self-pay	2,075 (1.7%)	708,490 (2.3%)	
	No charge	75 (0.1%)	62,940 (0.2%)	
	Other	2,725 (2.3%)	718,380 (2.3%)	
Total charge (inflation adjusted), median (IQR)		$107,104 ($60,222-$200,740)	$46,870 ($25,199-$88,593)	<0.001
Total cost, median (IQR)		$26,590 ($15,793-$46,878)	$11,686 ($6,785-$20,769)	<0.001
Comorbidities				
Hyperlipidemia		70,865 (59.4%)	19,186,065 (61.4%)	<0.001
Hypertension		33,880 (28.4%)	12,712,905 (40.7%)	<0.001
Obesity		22,435 (18.8%)	6,290,263 (20.1%)	<0.001
Smoker/tobacco user		51,115 (42.8%)	13,701,946 (43.8%)	0.003
Obstructive sleep apnea		11,320 (9.5%)	3,732,614 (11.9%)	<0.001
Hypothyroidism		13,285 (11.1%)	5,081,873 (16.3%)	<0.001
Immunocompromised		235 (0.2%)	55,285 (0.2%)	0.475
Anemia		9,930 (8.3%)	2,090,184 (6.7%)	<0.001
Pneumonia		5,860 (4.9%)	2,880,509 (9.2%)	<0.001
Liver disease		1,400 (1.2%)	825,755 (2.6%)	<0.001
Pulmonary disease		27,130 (22.7%)	8,315,247 (26.6%)	<0.001
Prior MI		29,640 (24.8%)	7,450,643 (23.8%)	<0.001
Prior PCI		20,770 (17.4%)	6,787,963 (21.7%)	<0.001
Diabetes mellitus		91,655 (76.8%)	13,807,640 (44.2%)	<0.001

The majority of patients were white. The age distribution of CAD patients with and without LL amputation is shown in appendix 2. For comorbidities diabetes (76.8 vs. 44.2) anemia (8.3% vs. 6.7%), and prior myocardial infarction (24.8% vs. 23.8%) were higher in the coronary artery disease with amputation as compared to CAD only. Other comorbidities including hyperlipidemia (61.4% vs. 59.4%), hypertension (40.7% vs. 28.4%), obesity (20.1% vs. 18.8%), smoking/tobacco (43.8% vs. 42.8%), obstructive sleep apnea (11.9% vs. 9.5%), hypothyroidism (16.3% vs. 11.1%), pneumonia (9.2% vs. 4.9%), liver disease (2.6% vs. 1.2%), pulmonary disease (26.6% vs. 22.7%), and percutaneous intervention (21.7% vs. 17.4%) were more prevalent in the CAD-only arm.

Crude and propensity-matched outcomes

**Figure 3:**
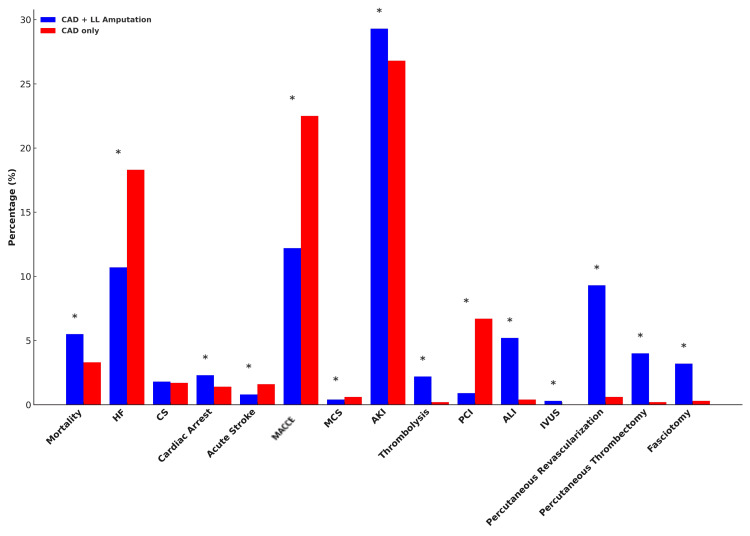
Bar diagram showing outcomes in respective groups. *P<0.05 was considered statistically significant. All figures are original to this study and created by the authors. HF: heart failure; CS: cardiogenic shock; MACCE: major adverse cardiac and cerebrovascular events; MCS: mechanical circulatory support; AKI: acute kidney injury; PCI: percutaneous coronary intervention; ALI: acute limb ischemia; IVUS: intravascular ultrasound; CAD: coronary artery disease; LL: lower limb

For crude outcomes, CAD with amputation arm had a higher onset of mortality (5.6% vs. 4.6%), cardiac arrest (2.3% vs. 1.7%), acute kidney injury (29.4% vs. 26.6%), thrombolysis (2.2% vs. 0.2%), acute limb ischemia (5.2% vs. 0.3%), need for lower limb percutaneous revascularization (9.2% vs. 0.6%), percutaneous thrombectomy (4% vs. 0.2%), and fasciotomy (3.2% vs. 0.3%) as compared to the CAD-only arm who had a higher onset of heart failure (18.8% vs. 10.6%), acute stroke (1.5% vs. 0.8%), MACCE (19.6% vs. 12.2%), need for mechanical circulatory support (0.7% vs. 0.5%), and PCI (5.7% vs. 0.9%). The two groups had no difference in the onset of cardiogenic shock (Table 2, Figure 3).

**Table 2:** Crude vs. propensity matched outcomes in CAD patients with and without LL amputation. HF: heart failure; CS: cardiogenic shock; MACCE: major adverse cardiac and cerebrovascular events; MCS: mechanical circulatory support; AKI: acute kidney injury; PCI: percutaneous coronary intervention; ALI: acute limb ischemia; IVUS: intravascular ultrasound; CAD: coronary artery disease; LL: lower limb Propensity scores were generated from a multivariable logistic regression model that included covariates such as baseline characteristics and medical comorbidities. A 1:1 matching algorithm was used with a caliper width of 0.2 times the SD of the logit of the propensity score.

Outcome	Crude		p-Value	Propensity matched		p-Value
	CAD+LL amputation	CAD only		CAD+LL amputation	CAD only	
N	119,320 (0.4%)	31,260,619 (99.6%)	-	23,261 (50.0%)	23,261 (50.0%)	-
Mortality	6,655 (5.6%)	1,426,649 (4.6%)	<0.001	1,285 (5.5%)	771 (3.3%)	<0.001
HF	12,705 (10.6%)	5,864,373 (18.8%)	<0.001	2,481 (10.7%)	4,260 (18.3%)	<0.001
CS	2,100 (1.8%)	559,140 (1.8%)	0.742	408 (1.8%)	385 (1.7%)	0.410
Cardiac arrest	2,725 (2.3%)	543,805 (1.7%)	<0.001	527 (2.3%)	327 (1.4%)	<0.001
Acute stroke	930 (0.8%)	466,565 (1.5%)	<0.001	183 (0.8%)	379 (1.6%)	<0.001
MACCE	14,605 (12.2%)	6,116,398 (19.6%)	<0.001	2,834 (12.2%)	5,228 (22.5%)	<0.001
MCS	540 (0.5%)	217,590 (0.7%)	<0.001	103 (0.4%)	149 (0.6%)	0.004
AKI	35,105 (29.4%)	8,324,052 (26.6%)	<0.001	6,818 (29.3%)	6,239 (26.8%)	<0.001
Thrombolysis	2,615 (2.2%)	57,200 (0.2%)	<0.001	507 (2.2%)	35 (0.2%)	<0.001
PCI	1,125 (0.9%)	1,603,355 (5.1%)	<0.001	219 (0.9%)	1,568 (6.7%)	<0.001
Lower limb outcomes						
ALI	6,190 (5.2%)	103,335 (0.3%)	<0.001	1,206 (5.2%)	89 (0.4%)	<0.001
IVUS	325 (0.3%)	9,210 (⁓0.0%)	<0.001	62 (0.3%)	0 (0.0%)	<0.001
Percutaneous revascularization	11,020 (9.2%)	190,930 (0.6%)	<0.001	2,151 (9.3%)	139 (0.6%)	<0.001
Percutaneous thrombectomy	4,800 (4.0%)	70,950 (0.2%)	<0.001	938 (4.0%)	37 (0.2%)	<0.001
Fasciotomy	3,775 (3.2%)	66,540 (0.2%)	<0.001	732 (3.2%)	64 (0.3%)	<0.001

Our analysis revealed similar results on a propensity-matched analysis revealing CAD with amputation arm had a higher onset of mortality (5.5% vs. 3.3%), cardiac arrest (2.3% vs. 1.4%), acute kidney injury (29.3% vs. 26.8%), thrombolysis (2.2% vs. 0.2%), acute limb ischemia (5.2% vs. 0.4%), need for percutaneous revascularization (9.3% vs. 0.6%), lower limb percutaneous thrombectomy (4% vs. 0.2%) and fasciotomy (3.2% vs. 0.3%) as compared to the CAD-only arm who had a higher onset of heart failure (18.3% vs. 10.7%), acute stroke (1.6% vs. 0.8%), MACCE (22.5% vs. 12.2%), need for mechanical circulatory support (0.6% vs. 0.4%), PCI (6.7% vs. 0.9%). Appendix 3 represents a balance plot of patient groups after propensity score matching.

Outcomes after multivariable logistic regression

Given the difference in the number of patients in both groups, we performed a multivariate analysis that confirmed a higher odds ratio of mortality (OR: 1.24, CI: 1.17-1.31), cardiac arrest (OR: 1.10, CI: 1.01-1.20), acute kidney injury (OR: 1.04, CI: 1.01-1.07), thrombolysis (OR: 11.29, CI: 10.30-12.37), acute limb ischemia (OR: 15.83, CI: 14.85-16.87), percutaneous revascularization (OR: 14.72, CI: 14.02-15.45), percutaneous thrombectomy (OR: 16.09, CI: 14.97-17.29), and fasciotomy (OR: 13.93, CI: 12.89-15.05) in the CAD with amputation arm as compared to the CAD-only arm. The multivariate analysis also revealed a lower incidence of heart failure (OR: 0.45, CI: 0.43-0.47), cardiogenic shock (OR: 0.79, CI: 0.71-0.87), acute stroke (OR: 0.54, CI: 0.47-0.62), MACCE (OR: 0.52, CI: 0.50-0.54), need for mechanical circulatory support (MCS) (OR: 0.50, CI: 0.41-0.61), and need for percutaneous intervention (PCI) (OR: 0.16, CI: 0.14-0.18). The results of the multivariate analysis are presented in Table 3.

**Table 3:** Multivariate analysis using logistic regression comparing outcomes in CAD+LL amputation and CAD only as a reference. OR: odds ratio; HF: heart failure; CS: cardiogenic shock; MACCE: major adverse cardiac and cerebrovascular events; MCS: mechanical circulatory support; AKI: acute kidney injury; PCI: percutaneous coronary intervention; ALI: acute limb ischemia; IVUS: intravascular ultrasound; CAD: coronary artery disease; LL: lower limb A two-staged multivariable mixed-effects logistic regression was employed incorporating patient-level variables, medical comorbidities, and socioeconomic status, as well as hospital-level variables in the analysis.

Outcomes	Adjusted OR	95% CI	p-Value
Mortality	1.24	1.17-1.31	<0.001
HF	0.45	0.43-0.47	<0.001
CS	0.79	0.71-0.87	<0.001
Cardiac arrest	1.10	1.01-1.20	0.039
Acute stroke	0.54	0.47-0.62	<0.001
MACCE	0.52	0.50-0.54	<0.001
MCS	0.50	0.41-0.61	<0.001
AKI	1.04	1.01-1.07	0.023
PCI	0.16	0.14-0.18	<0.001
Lower limb outcomes			
ALI	15.83	14.85-16.87	<0.001
IVUS	8.52	6.57-11.05	<0.001
Percutaneous revascularization	14.72	14.02-15.45	<0.001
Percutaneous thrombectomy	16.09	14.97-17.29	<0.001
Thrombolysis	11.29	10.30-12.37	<0.001
Fasciotomy	13.93	12.89-15.05	<0.001

**Figure 4:**
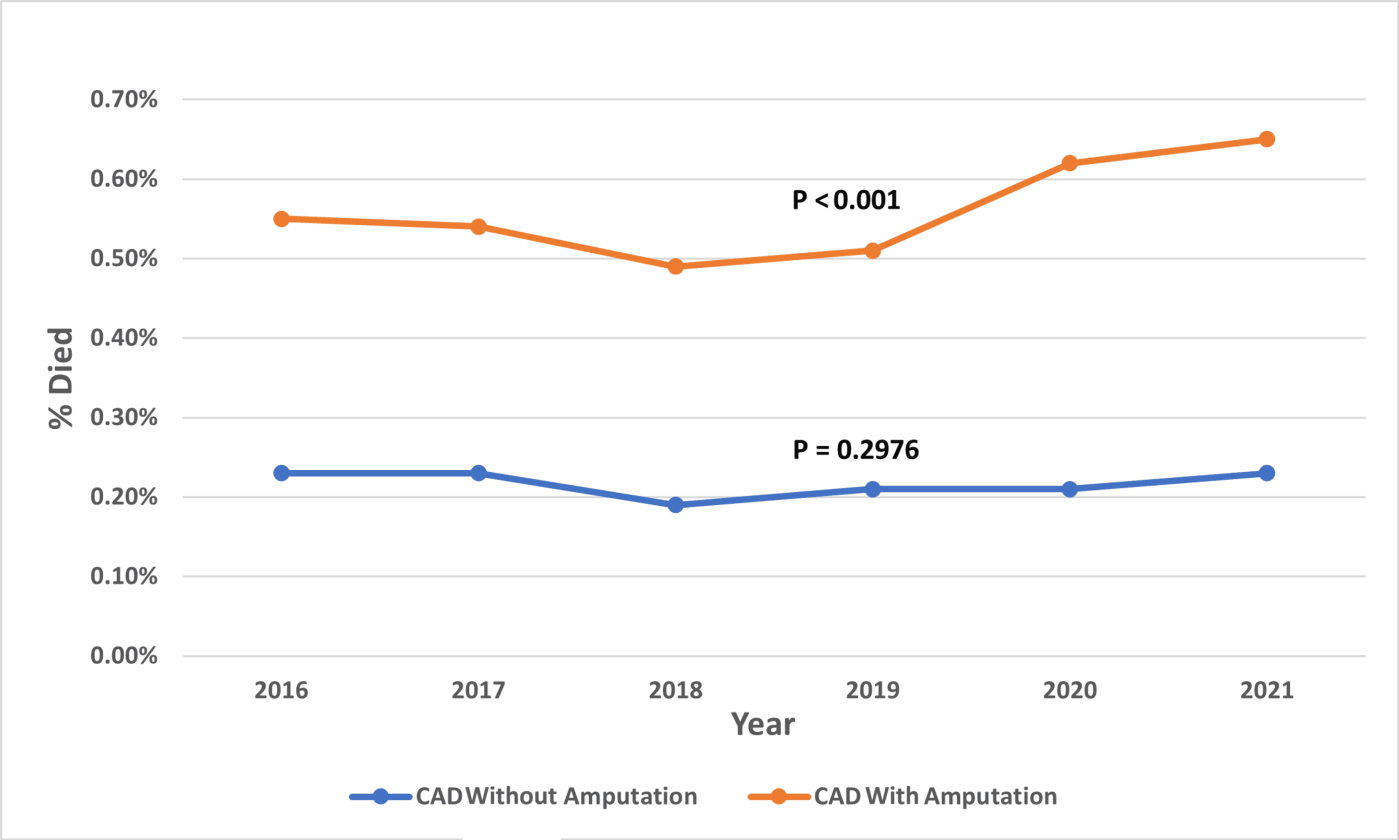
Trend of mortality among CAD patients undergoing lower limb amputation from 2016 to 2021. All figures are original to this study and created by the authors. CAD: coronary artery disease

Furthermore, our findings indicate an increasing trend in mortality from 2016 to 2021 among patients with CAD undergoing lower limb amputation (Figure 4).

Resource utilization

With regards to healthcare utilization, the length of stay was longer in patients with coronary artery disease and amputation (10 days) as compared to four days in the CAD-only arm. Most of the patients (45.8% vs. 40.3%) were admitted to the hospitals in the southern region. Total charges were $26,590 ($15,793-$46,878) in the amputation arm compared to $11,686 ($6,785-$20,769) in CAD only. Most hospital admissions were in urban teaching settings and larger hospitals in both groups with Medicare insurance.

## Discussion

Heart disease is reported to be associated with increased mortality among patients with amputation [24,25]. The high cardiac-related mortality after amputation has been investigated in previous cohort studies in the United States [26,27]. However, studies assessing short-term in-hospital outcomes in these patients are limited. This nationally representative study aimed to compare inpatient outcomes, comorbidities, and healthcare utilization between patients with coronary artery disease (CAD) who underwent lower limb amputation and those with CAD only.

The main findings of the present study may be summarized as follows: (1) patients with CAD and lower limb amputations experience significantly worse outcomes, including higher mortality rates and cardiac arrest, and (2) patients with amputation and CAD had higher healthcare resource utilization, including longer hospital stays and increased total adjusted hospital charges. These results were consistent across both multivariate and propensity score matching analyses.

Previous research has also reported the association between CAD and poor outcomes in patients with amputations. A meta-analysis by Stern et al. in 2017 found at least a two-fold increase in mortality for patients with amputations when CAD was a comorbid condition [7]. Additionally, a multicenter study (Reduction of Atherothrombosis for Continued Health {REACH} Registry) by Abola et al. in 2012 observed that patients with peripheral artery disease (PAD) complicated by amputation had nearly twice the rate of cardiovascular death and all-cause mortality compared to PAD patients without amputations [28]. In 2020, Fard et al. conducted an observational study involving 382 patients and found that those who underwent lower limb amputation had a high mortality rate within the first year. The study identified myocardial infarction as an independent factor associated with one-year mortality, with an odds ratio (OR) of 1.7 [29]. It has been shown that cardiovascular disease decreases the likelihood of becoming a prosthetic walker and negatively impacts mobility outcomes following lower limb amputation [24]. Our findings indicate an increasing trend in mortality from 2016 to 2021 among patients with CAD undergoing lower limb amputation. The increasing mortality trend may be attributed to an aging population, rising comorbidities, changes in revascularization strategies, and potential disruptions in care delivery during the COVID-19 pandemic.

Interestingly, diabetes prevalence was higher in the CAD with lower limb amputation group compared to the CAD-only group, whereas other comorbidities known to increase atherosclerotic burden, such as hyperlipidemia, hypertension, smoking, and prior PCI, were more common in the CAD-only group. Additionally, heart failure, MCS, and MACCE were higher in the CAD-only arm. This may be explained by the fact that the etiology leading to amputation in patients with CAD is primarily driven by peripheral neuropathy and microvascular damage associated with diabetes [30]. The lower MACCE and PCI rates in amputees may indicate less aggressive coronary management due to higher comorbid burden. Furthermore, this may also reflect differences in baseline cardiovascular disease severity, lower rates of coronary interventions, or higher non-cardiac mortality limiting the detection of these outcomes. While this result is statistically significant, it is less clinically impactful. Despite propensity-score matching minimizing selection bias, residual unmeasured confounders such as disease severity and frailty may still influence outcomes.

Our results indicate a higher risk of lower limb complications, including acute limb ischemia and the need for interventional procedures such as IVUS, percutaneous thrombectomy, and fasciotomy. Compromised vascular health and systemic inflammation in patients with underlying cardiovascular diseases exacerbate ischemic events and thrombotic risks, necessitating advanced interventions to manage severe complications. Previous studies have shown a higher prevalence of CAD in patients with critical limb ischemia who undergo major amputation [31].

This study has found anemia to be more prevalent among patients with coronary artery disease (CAD) who undergo amputations. A prospective cohort study by Desormais et al. revealed that 50.9% of patients hospitalized for peripheral artery disease (PAD) were anemic, and this condition was significantly linked to increased risks of death and major amputation. The study reported a hazard ratio (HR) of 1.44 (95% CI: 1.15-1.80) for amputation in anemic patients, highlighting a notable rise in risk [32]. Similarly, a large database analysis by Lüders et al. demonstrated that acute anemia is associated with a 6.4-fold increase in in-hospital mortality among PAD patients (p<0.001) [33]. Patients with CAD and amputations have worse outcomes due to several interrelated factors, including an increased atherosclerotic burden, as evidenced by higher coronary artery calcification scores (CACS) in amputees compared to controls [25]. Also, reduced mobility and functional decline post-amputation might also contribute to worse cardiovascular outcomes, as highlighted by the American Heart Association [34]. This study demonstrated greater healthcare resource utilization in the amputation group. The longer hospital stays and higher total charges in the amputation group likely reflect greater procedural complexity, higher rates of complications such as acute limb ischemia, fasciotomy, and thrombectomy, and increased need for post-operative care and rehabilitation. These differences are both statistically and clinically significant, underscoring the substantial economic burden of CAD patients requiring amputation. Regional disparities were evident, with higher admissions in the southern region and urban teaching hospitals, possibly due to higher prevalence of CAD and PAD, concentration of specialized vascular services, and greater access to tertiary care centers. These findings highlight resource allocation challenges, suggesting that urban teaching hospitals, which already manage a disproportionate share of complex cases, may require additional funding and staffing to accommodate high-cost patients. Policymakers should consider cost-management strategies that focus on early PAD screening, limb preservation programs, and post-discharge rehabilitation access to reduce hospitalization costs and prevent disease progression, particularly in high-burden regions. The predominance of Medicare insurance reflects an older, high-risk population with multiple comorbidities, emphasizing the economic burden on government-funded healthcare and the need for targeted preventive strategies.

Limitations

This study has several limitations inherent to the NIS database. First, NIS is a discharge-level database, meaning multiple admissions for the same patient are recorded as separate entries, preventing longitudinal follow-up or assessment of readmissions. Second, reliance on ICD-10-CM codes introduces the potential for coding errors or misclassification bias, which may affect case identification and outcome accuracy. Additionally, the database lacks detailed clinical variables such as laboratory values, hemodynamic parameters, and medication use, limiting granular risk stratification [35]. Furthermore, the etiology leading to amputations was not well classified, encompassing severe PAD to traumatic amputations.

## Conclusions

This study highlights the significant clinical burden associated with lower limb amputation in patients with CAD. The findings of this study demonstrate that amputation is associated with a higher rate of mortality and more extensive resource utilization in patients with CAD. These results emphasize the urgent need for improved CAD management in amputees, particularly through early detection and aggressive PAD treatment to prevent disease progression. Targeted preventive strategies should include routine ankle-brachial index screening, intensive lipid-lowering therapy, glycemic control in diabetics, structured exercise therapy, and smoking cessation programs to reduce the risk of critical limb ischemia. Optimized perioperative care could involve preoperative cardiovascular risk stratification (e.g., echocardiography, coronary CT angiography), perioperative hemodynamic monitoring, and thromboprophylaxis to improve surgical outcomes. Post-discharge care should prioritize early rehabilitation, multidisciplinary cardiovascular follow-up, and remote monitoring for complications to reduce hospital readmissions. Future research should explore the role of advanced cardiovascular imaging (e.g., CT fractional flow reserve, intravascular ultrasound) in refining CAD risk assessment in amputees, as well as novel endovascular and pharmacologic interventions to mitigate PAD-related complications.

## Figures and Tables

**Figure 5:**
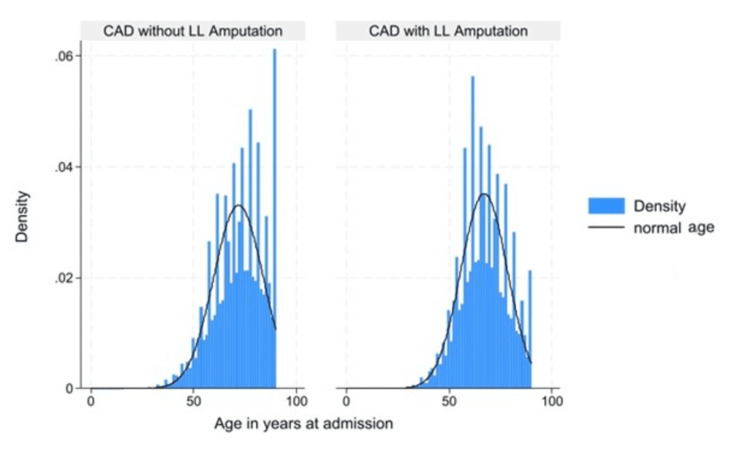
Age distribution at admission for CAD patients with and without LL amputation. CAD: coronary artery disease; LL: lower limb

**Figure 6:**
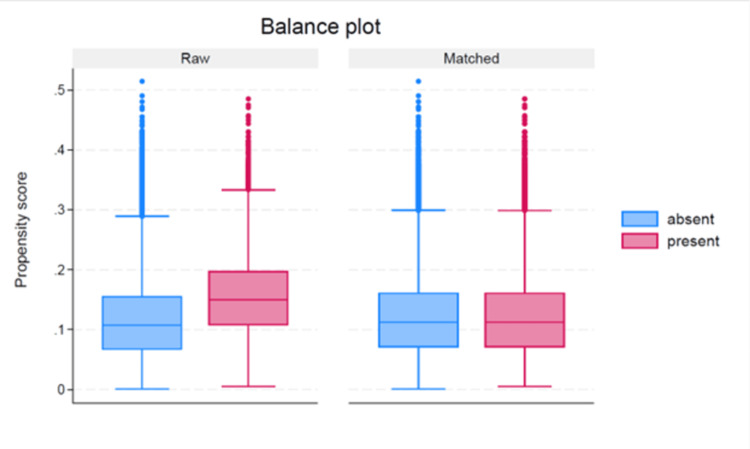
Balance plot for propensity matching in CAD patients with and without LL amputation. CAD: coronary artery disease; LL: lower limb

## Appendices

Appendix 1

**Table 4:** ICD-10-CM codes for included diagnoses and procedures. ICD-10-CM: International Classification of Diseases, 10th Revision, Clinical Modification

Variable	ICD-10-CM code
Coronary artery disease	I251, I2510, I2511, I25110, I25111, I25118, I25119
Lower limb amputation	0Y620ZZ, 0Y630ZZ, 0Y640ZZ, 0Y670ZZ, 0Y680ZZ, 0Y6C0Z1-0Y6C0Z3, 0Y6D0Z1-0Y6D0Z3, 0Y6F0ZZ, 0Y6G0ZZ, 0Y6G0Z1-0Y6G0Z3, 0Y6J0Z1-0Y6J0Z3

**Table 5:** ICD-10-CM codes for included outcomes. ICD-10-CM: International Classification of Diseases, 10th Revision, Clinical Modification

Variable	ICD-10-CM code
Acute limb ischemia	I74-I74.9
Fasciotomy	0J8L0ZZ, 0J8M0ZZ, 0J8N0ZZ, 0J8P0ZZ, 0JDL0ZZ, 0JDM0ZZ, 0JDN0ZZ, 0JDP0ZZ, 0JNL0ZZ, 0JNM0ZZ, 0JNN0ZZ, 0JNP0ZZ, 0JBL0ZZ, 0JBM0ZZ, 0JBN0ZZ, 0JBP0ZZ
Intravascular ultrasound	B54CZZ3, B54BZZ3, B54DZZ3
Percutaneous thrombectomy	04CC3ZZ, 04CC4ZZ, 04CD3ZZ, 04CD4ZZ, 04CH3ZZ, 04CH4ZZ, 04CJ3ZZ, 04CJ4ZZ, 04CK3ZZ, 04CK4ZZ, 04CL3ZZ, 04CL4ZZ, 04CM3ZZ, 04CM4ZZ, 04CN3ZZ, 04CN4ZZ, 04CP3ZZ, 04CP4ZZ, 04CQ3ZZ, 04CQ4ZZ, 04CR3ZZ, 04CR4ZZ, 04CS3ZZ, 04CS4ZZ, 04CT3ZZ, 04CT4ZZ, 04CU3ZZ, 04CU4ZZ, 04CV3ZZ, 04CV4ZZ, 04CW3ZZ, 04CW4ZZ, 04CY3ZZ, 04CY4ZZ
Thrombolysis	3E05017, 3E05317, 3E06017, 3E06317
Lower limb revascularization	047C.XXX, 047D.XXX, 047H.XXX, 047J.XXX, 047K.XXX, 047L.XXX, 047M.XXX, 047N.XXX, 047P.XXX, 047Q.XXX, 047R.XXX, 047S.XXX, 047T.XXX, 047U.XXX, 047V.XXX, 047W.XXX, 047Y.XXX
Percutaneous coronary intervention	027034Z, 027134Z, 027234Z, 027334Z, 02703DZ, 02713DZ, 02723DZ, 02733DZ, 02703TZ, 02713TZ, 02723TZ, 02733TZ, 0270346, 0271346, 0272346, 0273346, 02703D6, 02713D6, 02723D6, 02733D6, 02703T6, 02713T6, 02723T6, 02733T6
Myocardial infarction	I2101, I2102, I2109, I2111, I2119, I2121, I2129, I213, I214
Acute heart failure	I5021, I5023, I5031, I5033, I5041, I5043, I50811, I50813
Acute stroke	I639, I638, I6359, I63549, I63219, I63212, I63211, I6320, I6309, I63039, I63032, I63031, I6302, I63019, I63012, I63011, I6300
Sepsis	A400, A401, A410, A411, A412, A413, A414, A415, A418, A419
Pulmonary embolism	I26.XX
Cardiogenic shock	R570
Sudden cardiac arrest	I46
Mechanical circulatory support	5A02110, 5A02210, 5A0211D, 02HA3RZ, 5A02116, 5A0221D, 5A1522F, 5A1522G, 5A1522H, 5A15A2F, 5A15A2G, 5A15A2H, 5A15223
Pacemaker implantation	0JH604Z, 0JH605Z, 0JH606Z

**Table 6:** ICD-10-CM codes for cohort identification and stratification with major baseline comorbidities. ICD-10-CM: International Classification of Diseases, 10th Revision, Clinical Modification

Variables	ICD-10-CM codes
Hypertension	I10, I1150, I1151, I1152, I1158, I1159
Hyperlipidemia	E785, E7889, E7800, E781, E782, E783, E784, E784, E780, E7801, Q998, Z8639
Active smoking	F17, F172, F1720, F17200, F17201, F17203, F17208, F17209, F1721, F17210, F17211, F17213, F17218, F17219, F1722, F17220, F17221, F17223, F17228, F17229, F1729, F17290, F17291, F17293, F17298, F17299, Z87891
Obesity	E66.0, E66.1, E66.2, E66.3, E66.8, E66.9
Protein energy malnutrition	E40, E41, E42, E43, E44, E44.0, E44.1, E45, E46
Pneumonia	J12.0, J12.1, J12.2, J12.3, J12.8, J12.9, J13, J14, J15, J15.0, J15.1, J15.2, J15.3, J15.4, J15.4, J15.5, J15.5, J15.6, J15.7, J15.8, J15.8 J16, J17, J18.0, J18.1, J18.2, J18.8, J18.9
Liver disease	K70.0, K70.1, K70.2, K70.3, K70.4, K70.9, K71.0, K71.1, K71.2, K71.3, K71.4, K71.5, K71.6, K71.7, K71.8, K71.9, K72.0, K72.1, K72.9, K73.0, K73.1, K73.2, K73.8, K73.9, K74.0, K74.1, K74.2, K74.3, K74.4, K74.5, K74.6, K75.0, K75.1, K75.2, K75.3, K75.4, K75.8, K75.9, K76.0, K76.1, K76.2, K76.3, K76.4, K76.5, K76.6, K76.7, K76.8, K76.9, K77
Complicated diabetes	E10.6, E10.7, E11.6, E11.7, E13.6, E13.7, E11.21, E11.22, E11.42, E11.51
Uncomplicated diabetes	E11.9, E10.9, E13.9
Chronic kidney disease	N18.1, N18.2, N18.3, N18.30, N18.31, N18.32, N18.4, N18.5, N18.6, N18.9
Peptic ulcer disease excluding bleeding	K25.3, K26.3, K27.3, K28.3
Psychosis	F20.0, F20.1, F20.2, F20.3, F20.5, F20.8, F20.9, F22, F23, F25.0, F25.1, F25.2, F25.8, F25.9, F10.5, F11.5, F12.5, F13.5, F14.5, F15.5, F16.5, F19.5, F06.0, F06.2, F06.3, F28, F29
Depression	F32.0, F32.1, F32.2, F32.3, F32.4, F32.5, F32.8, F32.9, F33.0, F33.1, F33.2, F33.3, F33.4, F33.8, F33.9
Pulmonary disease	J449, J444, J440, J441
Hypothyroidism	E02, E30, E31, E32, E33, E34, E35, E36, E37, E38, E39
Anemia	D501, D508, D509, D500, D510, D511, D512, D513, D518, D519, D520, D528, D529, D53, D530, D531, D532, D538, D539
Valvular disease	I058, I059, I060, I061, I062, I069, I070, I071, I072, I078, I080, I081, I082, I083, I088, I089
Pulmonary circulation disorders	I2601, I2602, I2609, I2690, I2692, I2693
Renal failure	N170, N171, N172, N178, N179, N181, N182, N183, N1830, N1831, N1832, N184, N185, N186, N189, N19, N200, N201, N202, N209, N210, N211, N218, N219, N22
Fluid and electrolyte disorders	E860, E861, E869, E870, E871, E872, E873, E874, E875, E876, E8770, E8771, E8779, E878
Alcohol abuse	F1010, F1011, F10120, F10121, F10129, F10130, F10131, F10132, F10139, F1014, F10150, F10159, F10180, F10181, F10182, F10188, F1019, F1020, F1021, F10220, F101221, F10229, F10230, F10231, F10232, F10239, F1024, F10250, F10251, F10259, F1026, F1027, F10280, F10281, F10282, F10288, F1029, F10920, F10921, F10929, F10930, F10939, F1094, F10950, F10951, F10959, F1096, F1097, F10980, F10981, F10982, F10988, F1099
Drug abuse	F1110, F1011, F1110, F1111, F1210, F1211, F1310, F1311, F140, F1411, F1510, F1511, F1610, F1611, F1810, F1811, F1910, F1911
Complicated hypertension	I150, I151, I152, I158, I159, I270. I272, I2720, I2721, I2722, I2723, I2729, I87311, I87312, I87313, I87319, I87321, I87322, I87323, I87329, I87331, I87332, I87333, I87339, I87391, I87392, I87393, I87399

**Table 7:** Definition of major outcomes. LOS: length of stay; AKI: acute kidney injury; MACCE: major adverse cardiac and cerebrovascular events; ECMO: extracorporeal membrane oxygenation; CAD: coronary artery disease

Outcomes	Definition
Mortality	All causes of death, including cardiovascular causes such as sudden cardiac death, death due to acute myocardial infarction, heart failure or cardiogenic shock, and non-cardiovascular causes
AKI	Any acute kidney injury in stroke patients during the hospital stay
Cardiogenic shock	Shock resulting from primary failure of the heart in its pumping function
Sudden cardiac arrest	The sudden loss of all heart activity due to any type of arrhythmia in patients admitted with stroke
Myocardial infarction	Acute myocardial infarction in patients admitted with stroke
Cardiac arrhythmias	Development of cardiac arrhythmias in patients hospitalized for stroke
MACCE	Major adverse cardiac and cerebrovascular events in patients with stroke
Pulmonary embolism	Acute pulmonary embolism in patients hospitalized for stroke
Mechanical circulatory support	Any type of circulatory support with an intra-aortic balloon pump, impeller pump, ECMO, or external heart assist system utilization during the index hospitalization
Thrombolysis	Administration of thrombolytic agents for acute limb-threatening ischemia
Acute limb ischemia	Development of acute limb ischemia in patients admitted with CAD
Intravascular ultrasound	Patients with CAD undergoing lower extremity intravascular ultrasound
Percutaneous revascularization	Patients with CAD requiring percutaneous revascularization of lower extremity
Percutaneous thrombectomy	CAD patients undergoing percutaneous thrombectomy
Fasciotomy	CAD patients requiring lower extremity fasciotomy
LOS	The entire length of stay the patient spent in the hospital during the admission.
Cost of hospitalization	The total adjusted amount that the hospital got reimbursed for providing their services for the duration of hospitalization

**Table 8:** Variables used for propensity score matching.

Variables included in propensity matching
Age
Gender
Race
Rehab transfer
Elective/non-elective admissions
Hospital bed size
Hospital location and teaching status
Hospital region
Weekend admission
Payer
Hypertension
Obesity
Smoking
Pulmonary diseases
Hypothyroidism
Anemia
Liver disease
Valvular diseases
Pulmonary hypertension
Diabetes
Chronic kidney disease
Peptic ulcer disease
Fluid electrolyte imbalance
Drug abuse
Alcohol use disorder
Psychosis
Depression
Hyperlipidemia

**Table 9:** Matching variables used in multivariate logistics regression. HTN: hypertension; HLD: hyperlipidemia

Matching demographic variables
Year
Gender
Elective/non-elective
Hospital bed size
Hospital type (ownership, location, teaching status)
Hospital region
Transferred in/not transferred
Transferred out indicator
Admission day (weekend vs. weekday)
Payer type
HTN
Complicated hypertension
HLD
Obesity
Protein-energy malnutrition
Liver disease
Psychosis
Complicated diabetes
Uncomplicated diabetes
Depression
Chronic kidney disease
Smoker
Pulmonary disease
Hypothyroidism
Anemia
Valvular disease
Pulmonary circulation disorders
Renal failure
Peptic ulcer disease
Pneumonia
Fluid and electrolyte abnormalities
Alcohol abuse
Drug abuse

**Table 10:** Trend mortality and MACCE in CAD patients with vs. without LL amputation. MACCE: major adverse cardiac and cerebrovascular events; CAD: coronary artery disease; LL: lower limb

Variables	CAD with amputation	CAD without amputation
Mortality	p=0.2976	p<0.001
2016	1225	180750
2017	1305	181760
2018	1165	177245
2019	1280	175485
2020	1300	214100
2021	1365	224540
MACCE	p<0.001	p<0.001
2016	3340	942684
2017	3700	957995
2018	2995	889650
2019	3095	883400
2020	2885	887905
2021	2995	882654

**Table 11:** Trends of LOS among CAD patients with and without LL amputation. LOS: length of stay; CAD: coronary artery disease; LL: lower limb

Years	CAD with amputation median length of stay (days)	IQR	CAD without amputation median length of stay (days)	IQR
2016	8	5-13	4	2-6
2017	8	5-13	4	2-6
2018	8	5-13	4	2-6
2019	8	5-13	4	2-6
2020	8	5-13	4	2-7
2021	8	5-13	4	2-7

**Table 12:** Comparison of the total costs of admissions in US dollars among CAD patients with and without LL amputation. CAD: coronary artery disease; LL: lower limb

Years	CAD with amputation median cost ($)	IQR	CAD without amputation median cost ($)	IQR
2016	74869	42269-135754	39694	21112-76809
2017	77644	43650-141915	40681	21676-78779
2018	77213	44036-140016	41762	22304-80814
2019	80574	45628-147680	43903	23499-84873
2020	84265	47255-152501	47386	25313-91795
2021	89577	50325-161482	50734	27164-98237

Appendix 2

Appendix 3
